# Combined ^1^H MRI, PET and Multinuclear MRS hybrid imaging system

**DOI:** 10.1186/2197-7364-1-S1-A6

**Published:** 2014-07-29

**Authors:** Janusz H Hankiewicz, Zbigniew Celinski, Kevin Smiley, Stan Majewski

**Affiliations:** UCCS Center for BioFrontiers Institute, University of Colorado at Colorado Springs, 1420 Austin Bluffs Parkway, CO 80918 Kragujevac, USA; Center for Advanced Imaging, Department of Radiology, West Virginia University, 1 Medical Center Drive, Morgantown, WV 26506 USA

In this abstract we describe a novel method of combining three imaging modalities: ^1^H Magnetic Resonance Imaging (MRI), Positron Emission Tomography (PET), and multinuclear Magnetic Resonance Spectroscopy (MRS) in one system dedicated for true molecular imaging.

The addition of PET to MRI was introduced to provide functional/metabolic information about diseases or natural processes in the human body. High resolution and soft tissue contrast of ^1^H MRI morphological images is complemented by PET’s ability to depict metabolic processes through the use of biologically active radioactively labeled imaging agents, paving the way for molecular imaging.

The application of localized ^1^H MRS is implemented by using the imaging coil tuned to proton resonances. Localized, or MRI-guided, MR spectroscopy enables quantification of chemical composition of different tissue components in small volumes of less than 0.1 ml. Particularly in the brain, ^1^H MRS can for example measure accurately N-acetyl aspartate (NAA), lactate, glutamate, creatine, and choline to provide a view of the progression of neuro-degeneration.

However, the use of coil exclusively tuned to ^1^H significantly limits the “window” of observation by elimination of other biologically relevant nuclei like ^13^C, ^14^N, ^17^O, or ^31^P. Therefore we propose introducing a second resonator that will be tuned to these nuclei. We anticipate that MRS of other than ^1^H nuclei will provide more spectroscopic details and remove the ambiguity that exists in the ^1^H spectra from overlapping lines. The additional coil tuned to specific X-nuclei can be embedded within the existing ^1^H coil or can be removable, to be added whenever MRS on these nuclei is deemed necessary.

